# Pushing the boundaries of neurosurgical oncology: evaluating the superiority of supratotal resection over gross total resection in intraoperative MRI-guided glioma surgery

**DOI:** 10.1007/s10143-025-03301-x

**Published:** 2025-02-06

**Authors:** Leyla Salimli Mirzayeva, Murat Uçar, Sümeyye Nur Budak, Ahmet Memduh Kaymaz, Nezih Yaylı

**Affiliations:** 1 Department of Radiology, Gazi University Faculty of Medicine, Ankara, Turkey; 2 Hacettepe University Department of Public Health, Ankara, Turkey; 3 Department of Neurosurgery, Gazi University Faculty of Medicine, Ankara, Turkey

**Keywords:** Intraoperative magnetic resonance imaging, Supratotal resection, Gross total resection, Glioma, Survival, Neurosurgery, Neurooncology

## Abstract

Using intraoperative MRI (iMRI) in glioma surgery can enhance the extent of resection (EOR) and improve survival rates for patients diagnosed with low grade gliomas (LGG) or high grade gliomas (HGG). This study focused on patients who underwent iMRI-guided surgery for LGG and HGG at our center. Our objective was to compare the patient survival time and recurrence rate between supratotal resection (SpTR) and gross total resection(GTR). To the best of our knowledge, this is the first study comparing SpTR with GTR using iMRI in glioma surgery. This study included 71 patients who had undergone iMRI-guided GTR and SpTR. The volume of the tumors was measured using postcontrast 3D T1W series and 3D FLAIR series taken the day before surgery, and the volume of the operation cavity was calculated from iMRI images. The effects of SpTR and GTR on overall and progression-free survival (OS and PFS) were analyzed by the log-rank test using Kaplan‒Meier curves. The associations between the extent of resection and tumor grade, and between recurrence and tumor grade were examined using the chi-square test. The rate of recurrence in patients diagnosed with HGG was greater than that in patients diagnosed with LGG (*p* = 0.022). While patients who received SpTR had a greater OS time (105.9 months) than did those who underwent GTR (92.8 months), the difference was not statistically significant. The patients with LGG had a significantly longer PFS time than did the patients with HGG (86.5 ± 5.9 months, 95% CI = 74.9–98.2, *p* < 0.0001). Of 23 patients diagnosed with HGG, SpTR was achieved in 9 and GTR was achieved in 14. The median OS time was longer in patients who underwent SpTR than in those who underwent GTR, but there was no statistically significant difference [101.2 ± 20.5 months (95% CI: 80.7–121.7) vs. 70.6 ± 9.9 (95% CI: 60.7–80.5) *p* = 0.9]. Neurosurgeons are increasingly choosing SpTR, especially in LGGs. Despite their slow growth, LGGs retain the potential for malignant transformation. This situation underscores the importance of maximum safe resection in the surgery of LGGs. iMRI-guided resection makes it easier for surgeons to show the EOR concurrent with the operation.

## Introduction

Gliomas are the most common type of primary brain tumor in adults. Despite the proven success of chemoradiotherapy, extended resection remains the most effective therapeutic technique for improving survival. Although emerging treatment methods such as immunotherapy and virotherapy are promising, they are not yet included in standardized treatment protocols since their effectiveness has not been fully proven. For this reason, the concept of supratotal resection (SpTR) has gained prominence in oncological neurosurgery, in addition to gross total resection (GTR). Studies have demonstrated that the SpTR of low-grade gliomas (LGG) can potentially delay malignant transformation [1]. In high-grade gliomas (HGG), the possibility of recurrence is reduced by safely expanding the resection margin beyond the contrast-enhancing area due to the infiltrative feature of the tumor [2].

Using intraoperative MRI (iMRI) in glioma surgery can enhance the extent of resection and improve survival rates for patients diagnosed with LGG or HGG [3]. The combination of iMRI and awake brain mapping helps minimize the risk of injury to subcortical pathways and allows for a greater extent of resection [4].

With this method, resection is carried out until functional boundaries are reached [5]. Functional boundaries might be located within, at the edge, or beyond the limits of the tumor. If a functional boundary is present inside the tumor mass, a subtotal or partial resection is carried out. GTR can be carried out if a functional boundary is identified at the tumor periphery. When functional margins are found beyond the tumor area, a part of the brain parenchyma that seems to be intact is removed along with the tumor, and this process is called SpTR [6].

During surgery for a space-occupying lesion in the brain, a parenchymal shift occurs because the increased intracranial pressure suddenly decreases during tumor excision. In this case, it becomes very difficult for the surgeon to distinguish the tumor boundaries with the bare eye. The use of iMRI eliminates this difficulty, and tumor boundaries can be clearly distinguished with iMRI, which is a non-invasive method, before reactive edema that may develop in the early postoperative period occurs.

This study focused on patients who underwent iMRI-guided surgery for LGG and HGG at our center. We specifically examined patients in whom SpTR and GTR were performed. Our objective was to compare the patient survival time and recurrence rate between these two resection rates. To the best of our knowledge, this is the first study comparing SpTR with GTR using iMRI in glioma surgery.

## Materials and methods

### Selection of patients

**Fig. 1:**
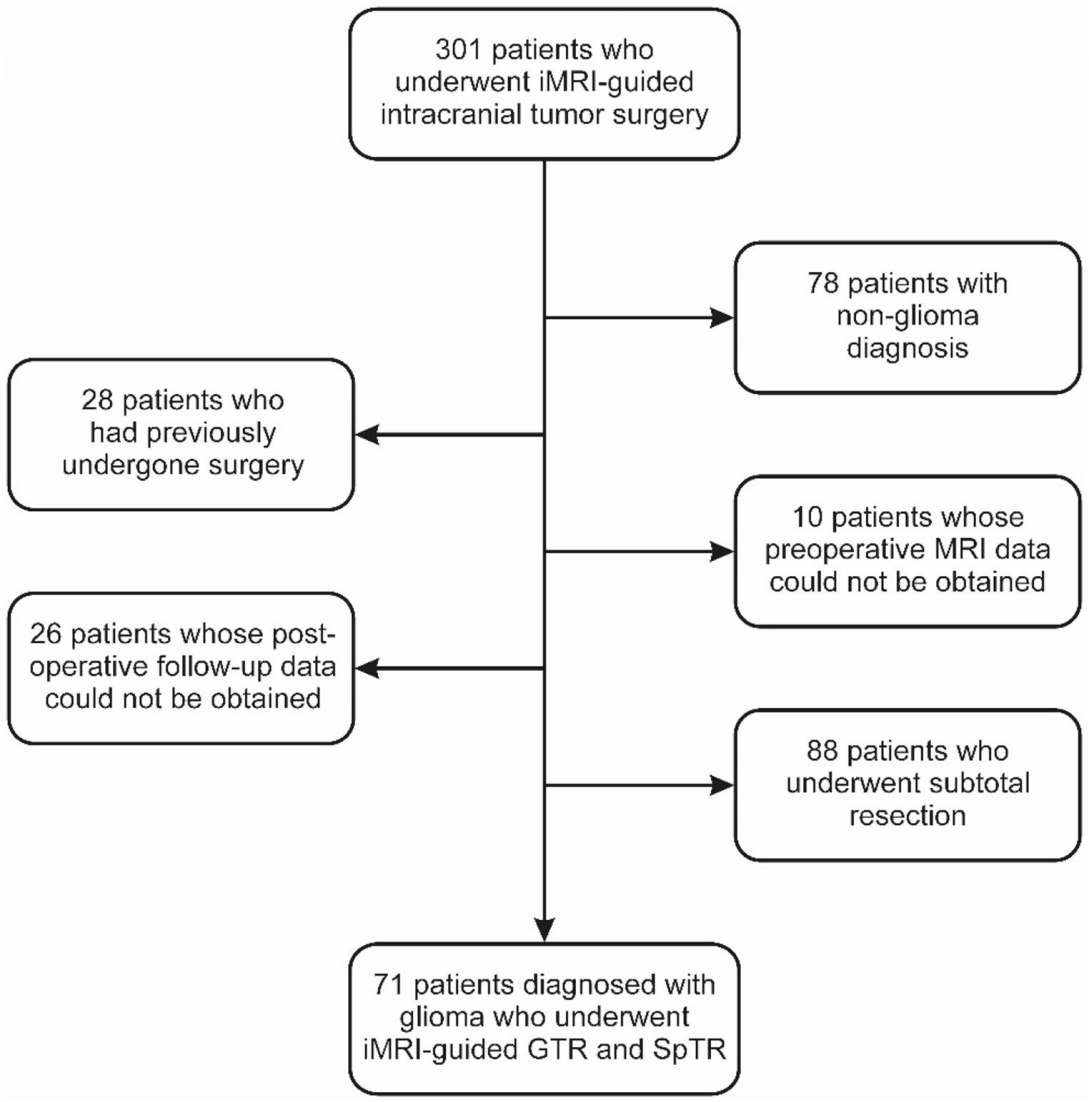
A flowchart shows the exclusion criteria in the study. iMRI: intraoperative magnetic resonance imaging; GTR: gross total resection; SpTR: supratotal resection

We conducted a retrospective analysis of 301 adult patients who underwent iMRI-guided intracranial tumor surgery at the department of neurosurgery of our institution, from January 2013 to January 2023. The study protocols adhered to the principles specified in the Helsinki Declaration. Patients who were diagnosed with conditions other than glioma (*n* = 78), patients who had previously undergone surgery (*n* = 28), patients whose preoperative MRI data could not be obtained (*n* = 10), patients whose postoperative follow-up data could not be obtained (*n* = 26), or patients who underwent subtotal resection (*n* = 88) were excluded from the study (Fig. 1). This study included a total of 71 patients who had undergone iMRI-guided GTR and SpTR. Data collection was approved by the Institutional Clinical Research Ethics Committee on 07.02.2022 (registration number: 99).

### Imaging

The patients underwent surgery in the operating room, which was conveniently connected to a separate room containing a 3 Tesla MRI device (Siemens Health Care Magnetom Verio, Erlangen, Germany) through a corridor. Following the administration of general anesthesia, a neuronavigation system (StealthStation^®^ Medtronic^®^, Inc., Minneapolis, USA) was established for all patients. The imaging protocol for iMRI consisted of a 3D T1-weighted volumetric sequence, a 3D FLAIR sequence, diffusion-weighted imaging with b values of 1000 s/mm2, and a T2-weighted gradient echo sequence (Table 1).

**Table 1:** IoMRI acquisition parameters

Sequence		TR (msec)	TE (msec)		Matrix		FOV (mm)		b Value	Slice thickness (mm)		Voxel size (mm)		Time (sec)
T2W (axial)		4200	107		256 × 256		220		-	4		0.7 × 0.5 × 4.0		245
T2 FLAIR		9000	85		256 × 256		220		-	4		1.1 × 0.9 × 4.0		236
DWI (axial)		4000	90		128 × 128		220		1000	4		0.8 × 0.8 × 4		125
3D T2 FLAIR		6000	420		256 × 256		230		-	0.9		0.8 × 0.8 × 0.8		266
Postcontrast T1 MPRAGE		1750	2.93		250 × 250		250		-	1		1.0 × 1.0 × 1.0		338

*Io MRI*, intraoperative magnetic resonance imaging; *TR*, repetition time; *TE*, echo time, *FOV*, field of view; *FLAIR*, fluid attenuated inversion recovery; *DWI*, Diffusion-weighted imaging; *MPRAGE*, magnetization-prepared rapid acquisition with gradient echo

### Surgical procedures

The main goal of the surgery was to achieve the highest possible resection while avoiding any neurological complications or sequelae. The procedure was performed under general anesthesia and, in certain situations, under a technique known as asleep-awake-asleep anesthesia. Through cortical mapping, the safe entrance zone was determined. The boundaries of the resection margins were established by performing subcortical mapping via intraoperative neurophysiology, a brain mapping technique. This technique helps identify the functional areas (motor, linguistic, tactile, visual, and cognitive) that define these borders.

### Volumetric analyses

The volume of the tumors was measured using postcontrast 3D T1W series and 3D FLAIR series taken the day before surgery, and the volume of the operation cavity was calculated from iMRI images. The measurements were manually taken using the editor module of 3D-Slicer version 5.0.2. (3D-Slicer is a free and open-source software program used for medical image calculation and visualization, specifically for volumetric analysis.) Measurement results were recorded in cubic centimeters.

### Statistical analyses

The Statistical Package for the Social Sciences was used for statistical analysis (version 22.0; SPSS, Inc., Chicago, IL). For continuous variables, the mean ± standard deviation (SD) or interquartile range (IQR) were given, whereas percentages were used for categorical data. The effects of SpTR and GTR on OS and PFS were analyzed by the log-rank test using Kaplan‒Meier curves. The associations between the EOR and tumor grade and between recurrence and tumor grade were examined using the chi-square test.

## Results

**Fig. 2:**
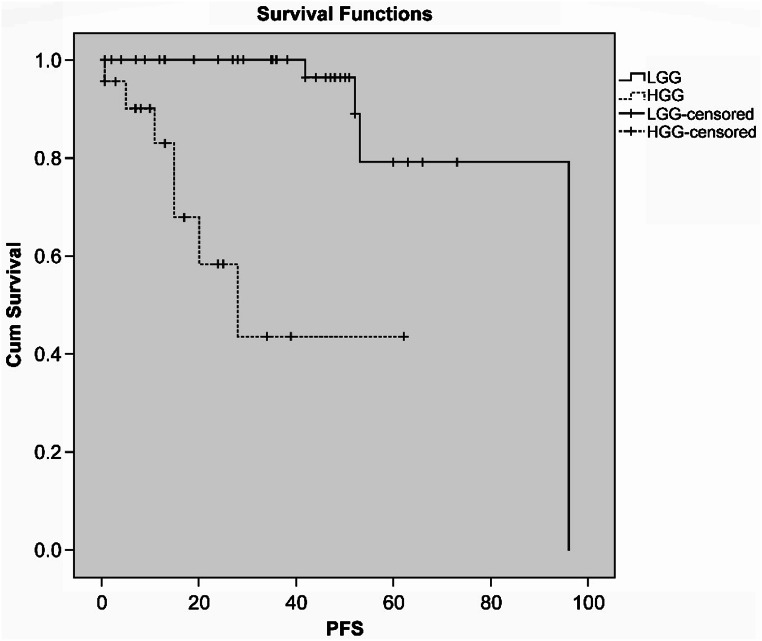
Kaplan-Meier curves show progression free survival rates in high grade glioma and low grade glioma patients. HGG: high grade glioma; LGG: low grade glioma; PFS: progression free survival

**Fig. 3:**
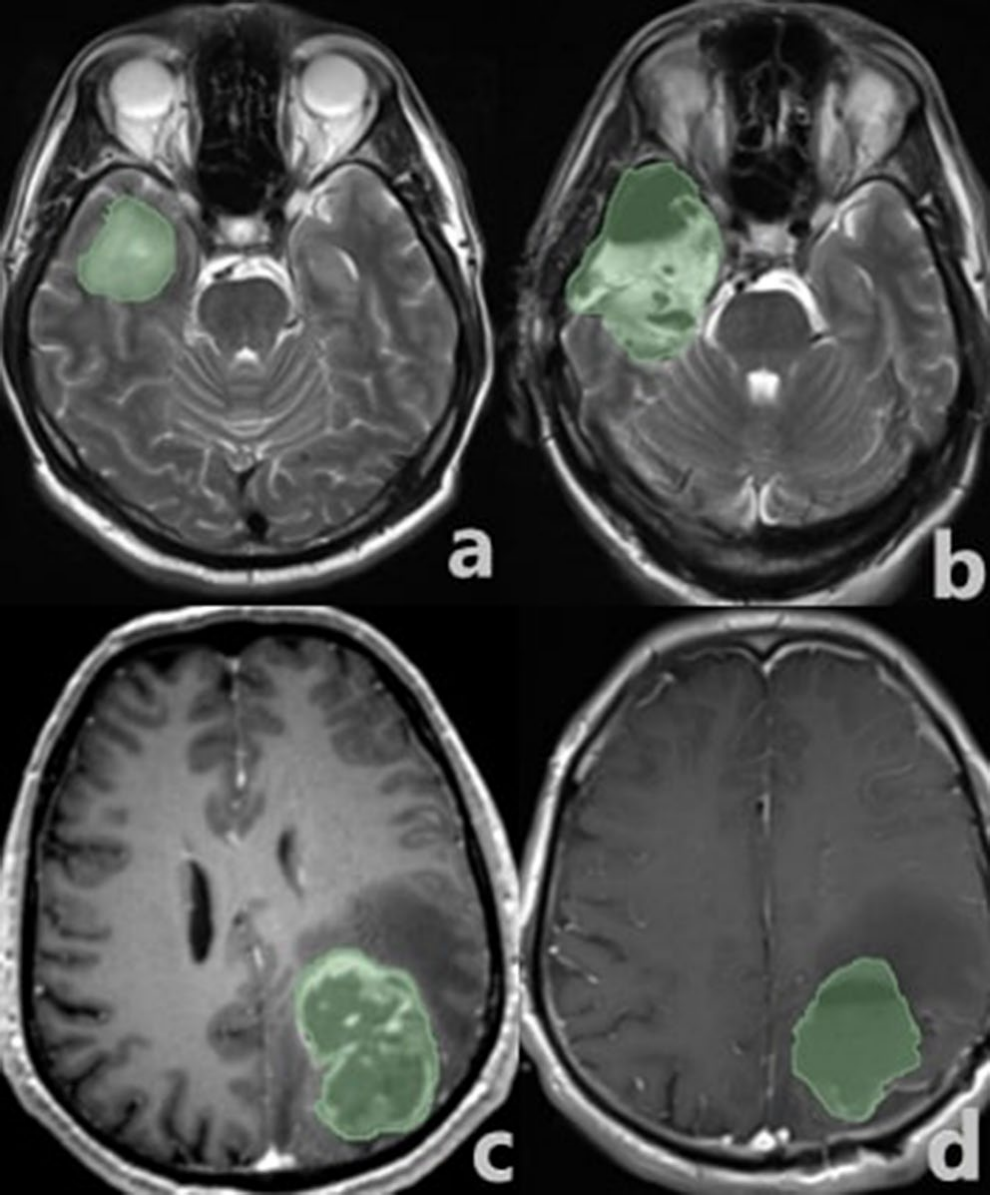
Axial T2W image shows preoperative volumetric analysis of the intraaxial mass located in the right temporal lobe (**a**); Volumetric analysis of the resection cavity in the intraoperative T2W MRI of the same case is compatible with supratotal resection (**b**); Preoperative axial postcontrast T1 image (**c**) shows volumetric analysis of the intraaxial mass in the left parieto-occipital; Volumetric analysis of the resection cavity in the intraoperative axial postcontrast T1W MRI of the same patient is compatible with gross total resection (**d**)

The patients included individuals aged 18–71 (median age 40.39 years). Thirty-two (45.1%) of these patients were female, and 39 (54.9%) were male. Although the number of tumors located in the frontal lobe was higher, no statistically significant difference was found between the groups (*p* = 0.3). No significant difference was found between the groups in gender and age data (*p* = 0.8 and *p* = 0.2, respectively). The histopathological diagnosis consisted of 48 patients with LGG and 23 patients with HGG. SpTR was detected in 33 patients, with 9 patients (27.3%) diagnosed with HGG and 24 patients (72.7%) diagnosed with LGG. GTR was obtained in 38 patients. Among these patients, 14 (36.8%) were diagnosed with HGG, and 24 (63.2%) were diagnosed with LGG. Tables 2 and 3 summarize the relationship between the EOR and histopathological type. Table 4 shows the detailed demographic and clinical outcome differences between the SpTR and GTR groups. During the 10-year follow-up period, recurrence was observed in a total of 11 patients, accounting for 15.5% of the study population. Of these 11 patients, 4 were initially diagnosed withLGG, and 7 were diagnosed with HGG. Among the 11 patients with recurrence, 7 had GTR (63.6%), and 4 had SpTR (36.4%). The rate of recurrence in patients diagnosed with HGG was greater than that in patients diagnosed with LGG (*p* = 0.022). While patients who received SpTR had a greaterOS time (105.9 months) than did those who underwent GTR (92.8 months), the difference was not statistically significant. The patients with LGG had a significantly longer PFS time than did the patients with HGG (86.5 ± 5.9 months, 95% CI = 74.9–98.2, *p* < 0.0001), as shown in Fig. 2. Figure 3 presents an example for GTR and SpTR, respectively, along with the calculation of the preoperative tumor and intraoperative cavity volume.

**Table 2:** EOR and OS times according to histopathological type

HGG	*n* = 23 (32.4%)	104.3 months	LGG	*n* = 48 (67.6%)	93.5 months
GTR	*n* = 14 (60.9%)	70.6 months	GTR	*n* = 24 (50%)	97.4 months
SpTR	*n* = 9 (39.1%)	101.2 months	SpTR	*n* = 24 (50%)	61.5 months

*EOR*: extent of resection; *OS*: overall survival; *HGG*: high grade glioma; *LGG*: low grade glioma; *GTR*: gross total resection; *SpTR*: supratotal resection

**Table 3:** Comparison of OS and PFS between SpTR and GTR

EOR	OS (month)		PFS (month)
SpTR	105.9	9 HGG 101.2 24 LGG 61.5	27.0 68.5
GTR	92.8	14 HGG 70.6 24 LGG 97.4	34.2 88.6

EOR: extent of resection; OS: overall survival; PFS: progression free survival; SpTR: supratotal resection; GTR: gross total resection

**Table 4:** Comparisons showing differences in demographic and clinical outcomes between the SpTR and GTR groups

EOR	Age	Gender	Localisation	Grade	IDH1 and 1p/19q status	Clinical Outcomes
GTR	18–70 (39.5 ± 15)	18 F, 20 M	Frontal 20 Parietal 7 Temporal 9 Occipital 1 Basal ganglia 1	HGG: 14 LGG: 24	IDH mutant: 12 IDH wild: 18 1p/19q codelesion: 3	A temporary neurological deficit in 1 case
SpTR	18–72 (40 ± 15.5)	14 F, 19 M	Frontal 19 Parietal 8 Temporal 3 Occipital 2 Infratentorial 1	HGG: 9 LGG: 24	IDH mutant: 11 IDH wild: 13 1p/19q codelesion: 4	Temporary neurological deficit in 5 cases, permanent neurological deficit in 2 cases

SpTR: supratotal resection; GTR: gross total resection; EOR: extent of resection; IDH: Isocitrate dehydrogenase; F: female; M: male

Of 23 patients diagnosed with HGG, SpTR was achieved in 9 and GTR was achieved in 14. The median OS time was longer in patients who underwent SpTR than in those who underwent GTR, but there was no statistically significant difference (Table 2). [101.2 ± 20.5 months (95% CI: 80.7–121.7) vs. 70.6 ± 9.9 (95% CI: 60.7–80.5) *p* = 0.9]. Recurrence was observed in five GTR cases and two SpTR cases, but there was no statistically significant difference between the PFS times [34.2 ± 8.4 months (95% CI: 25.8–42.6) vs. 27.0 ± 6.0 (95% CI: 21.0–33.0) *p* = 0.7].

Of 48 patients diagnosed with LGG, SpTR was achieved in 24 and GTR was achieved in 24. There was no statistically significant difference in OS times between these two patient groups. [61.5 ± 4.5 months (95% CI: 57.0–66.0) vs. 97.4 ± 7.4 (95% CI: 90.0–104.8) *P* = 0.6]. Recurrence was observed in 2 of the cases who underwent GTR and in 2 of the cases who underwent SpTR, but there was no statistically significant difference between the PFS times [88.6 ± 9.5 months (95% CI: 79.1–98.1) vs. 68.5 ± 2.9 (95% CI: 65.6–71.4) *p* = 0.9].

We reviewed the postoperative examination findings from the first week and the follow-up examination findings after discharge. In 63 (88.7%) out of 71 cases, no neurological deficit (ND) was observed in both postoperative and late periods. ND was detected in 8 (11.3%) cases, of which 6 (8.4%) were temporary and 2 (2.8%) were permanent. The EOR, histopathological grade and ND data of these cases are shown in the Table 5. Postoperative ND occurred more frequently in cases where SpTR was performed compared to cases with GTR (*p* = 0.02). However, the frequency of permanent ND did not show a significant change according to EOR (*p* = 0.21).

**Table 5:** EOR and histopathological grade information of cases developing postoperative ND

Cases	EOR	Histopathological grade	Neurological deficit	Duration
No.1	SpTR	LGG	Tinnitus and intermittent diplopia observed in the first postoperative month	Temporary
No.2	SpTR	LGG	Right temporal hemianopsia in a patient operated for left occipital LGG	Temporary
No.3	GTR	LGG	Dysphasia lasting 5 days after surgery	Temporary
No.4	SpTR	LGG	Weakness in the left upper and lower extremities lasting for 1 month after the surgery	Temporary
No.5	SpTR	LGG	Loss of strength in the right lower extremity (4/5)	Permanent
No.6	SpTR	LGG	Postoperative loss of strength in the right lower extremity (3/5)	Temporary
No.7	SpTR	HGG	Increased muscle weakness in the upper and lower extremities compared to preoperative examination	Temporary
No.8	SpTR	HGG	Right spastic hemiparesis	Permanent

EOR: extent of resection; SpTR: supratotal resection; GTR: gross total resection; LGG: low grade glioma; HGG: high grade glioma

In 23 cases diagnosed with HGG, the EOR was also classified according to the Response assessment in neuro-oncology (RANO) resection criteria. In the iMRI of 9 cases diagnosed with HGG who underwent SpTR, the contrast-enhancing (CE) tumor ratio was 0%, and the non-enhancing (non-CE) abnormal signal change ratio was < 5%, thus meeting the criteria for RANO class 1 (supramaximal resection).

In 14 HGG cases where GTR was performed, the ratio of CE tumor tissue was 0%, while the non-CE abnormal signal intensity change was < 5%. According to RANO resection criteria, these cases met class 2 A (complete CE resection). Since imaging was performed intraoperatively, surgery-related edema and gliosis at the resection margins had not yet developed. Therefore, if no residual tumor was left, abnormal signal intensity changes around the operation cavity are almost never seen on iMRI. For this reason, in the 14 HGG cases where GTR was performed, > 5% abnormal signal intensity changes were not observed in iMRI images.

## Discussion

Tumor recurrence is frequent in HGGs, even after GTR and standard chemoradiotherapy, mostly because of the infiltrative characteristics of the tumor. In the majority of cases, recurrence occurs within 2 cm of the resection margin. Compared with conventional procedures, SpTR is an innovative approach in glioma surgery that involves more comprehensive removal of the tumor. This procedure entails the extraction of more than 100% of the observable tumor tissue. Consequently, surgeons remove not only the tumor itself but also a portion of the adjacent healthy brain tissue.

We compared the recurrence and survival times by classifying the extent of resection on iMRI images of patients who underwent iMRI-guided glioma surgery at our center. To the best of our knowledge, no study has defined GTR or SpTR by measuring cavity volume on iMRI in glioma surgery. In previous studies, the volume of the surgical cavity was measured using either MRI taken within the first 24–72 h after surgery or MRI taken 2–3 months after surgery [7]. Operation cavity measurements made with iMRI are protected from possible shrinkage that may develop over time. Especially for occipitally located tumors, the volume of the operating cavity may change during the early postoperative period due to the effect of gravity. Therefore, more objective results are obtained with measurements made on iMRIs when evaluating SpTR and GTR.

While SpTR was applied in 24 (50%) of the 48 patients diagnosed with LGG included in our study, it could be applied in only 9 (39.1%) of the 23 patients diagnosed with HGG. There may be several reasons for this difference: (1) due to the poor response of LGGs to chemoradiotherapy compared to HGGs, the surgeon’s effort to remove normal parenchyma along with the tumor tissue may cause potential recurrence; (2) the proximity of HGGs to eloquent areas due to their infiltrative patterns, which prevents SpTR.

Our study revealed that patients who underwent SpTR had longer survival than did those who underwent GTR. However, it is important to note that we did not observe a significant difference between the two groups. SpTR was associated with a longer OS, especially when we compared SpTR with GTR in the case group diagnosed with HGG. There are publications in the literature showing that SpTR significantly increases survival time [7, 8, 9]. It has been reported that SpTR has a better effect on survival time, especially in LGG patients [9]. In addition to the aforementioned favorable outcomes, it is crucial to acknowledge that, compared with GTR, SpTR may be associated with an increased risk for postoperative neurological deficits and greater incidence of wound infection [11]. Therefore, it is essential to recognize that the decision on SpTR should be made individually, considering different patient- and tumor-specific criteria and with the advice of a team of qualified medical professionals.

The relationship between molecular biomarkers and the EOR in diffuse gliomas has also been shown in recent studies [12, 13]. While attaining GTR or even SpTR was essential for some molecular pathology subtypes, GTR increased the risk of surgical complications and offered no survival benefit for others [14]. Liaoning Li et al. conducted a study of the literature to provide comprehensive information for clinical and preclinical research on key molecular markers in the oncogenesis and progression of diffuse gliomas, as well as their guiding role in surgical treatment [12]. This review indicates that postoperative residual tumor tissue negatively affects OS in IDH-mutant type LGGs. It has been shown that SpTR is associated with longer OS times in both IDH-mutant and IDH wild-type LGGs [15]. In gliomas with 1p/19q codeletion and IDH mutation, extensive resection is associated with prolonged survival; however, a limiting factor for performing SpTR is the presence of the tumor in a functional or eloquent area [12, 13]. 

The literature has shown that maximal surgical resection improves survival in IDH-mutant type HGGs. However, the same outcome has not been achieved in IDH wild-type HGGs [16]. 

A postoperative transient ND after brain surgery is a short-term impairment in neurological function caused by the surgical process. These deficiencies are usually caused by transitory changes to brain tissue, blood flow, or nerve pathways during or immediately following surgery. They can be caused by inflammation, swelling (edema), mild injuries to neural structures, anesthetic effects, or brain manipulation during the surgery. Symptoms appear within the first week after surgery and can last from 12 h to several weeks [17]. Permanent NDs after brain surgery are often defined by symptoms such as persistent paresis (muscle weakness), dysphasia/aphasia (speech difficulty), or seizures lasting 4 weeks or more [18]. In our investigation, we observed that ND were more prevalent among patients who underwent SpTR during the postoperative follow-up period. However, a significant difference in the incidence of permanent ND over time was not found between the GTR and SpTR cohorts. The existing literature has conducted comparative analyses of early and long-term neuropsychological deficits between these two groups categorized by EOR. These studies have consistently demonstrated that SpTR is not associated with a higher incidence of sequelae compared to GTR [19]. These results support that the development of permanent sequelae is associated with damage to functional areas rather than the EOR.

Our study has several limitations. First, since we conducted a retrospective screening, our patient population was heterogeneous. Designing a nonblinded randomized trial for glioma surgery is quite challenging, and it is unlikely that there will be enough case balance to start a prospective randomized study for SpTR. Another limitations are the insufficient number of patients and lack of long term follow - up. The fact that this end point did not reach statistical significance might have been explained by the heterogeneous population, low size pool of patients and not enough follow up. Additionally, more in-depth evaluation of molecular markers is needed in current neuro-oncology standards.

## Conclusion

Tumor recurrence is common in gliomas even after GTR and standard chemoradiotherapy. SpTR is an innovative approach in glioma surgery that aims to extract more than 100% of observable tumor tissue, removing not only the tumor but also a portion of adjacent healthy brain tissue. In our study, while NDs were more frequently observed in patients who underwent SpTR during postoperative follow-up, no significant difference was identified between the GTR and SpTR groups regarding the development of permanent NDs over time. According to the literature, early and long-term neuropsychological deficits have been compared between these two EOR groups, and it has been demonstrated that SpTR is not linked to a higher incidence of sequelae than GTR [19]. 

Various studies have been conducted in the literature with cases using iMRI-guided glioma surgery, and evidence has been presented showing that iMRI positively affects tumor resection rates, OS, and PFS [20, 21]. To our knowledge, our study is the first to compare survival between patients who underwent SpTR or GTR using iMRI and to investigate the rate of recurrence during a 10-year follow-up. Despite their slow growth, LGGs retain the potential for malignant transformation. This situation underscores the importance of maximum safe resection in the surgery of LGGs [18]. 

## Data Availability

No datasets were generated or analysed during the current study.
